# Think zinc: Transient nutritional deficiency related to novel maternal *SLC30A2* mutation potentially precipitated by antenatal proton pump inhibitor exposure

**DOI:** 10.1002/ccr3.7213

**Published:** 2023-04-17

**Authors:** Emma Porter, Oonagh Molloy, Michelle Murphy, Cathal O'Connor

**Affiliations:** ^1^ Department of Dermatology South Infirmary Victoria University Hospital Cork Ireland; ^2^ Department of Medicine University College Cork Ireland; ^3^ INFANT Research Centre University College Cork Cork Ireland

**Keywords:** acrodermatitis, nutritional/metabolic dermatoses, zinc deficiency

## Abstract

A second‐born breastfed infant presented with zinc deficiency. His mother had a novel heterozygous mutation in *SLC30A2*. A previous baby did not have zinc deficiency but the mother had taken a proton pump inhibitor (PPI) during the second pregnancy. Antenatal PPI exposure may plausibly contribute to transient infantile zinc deficiency.

## Graphical abstract

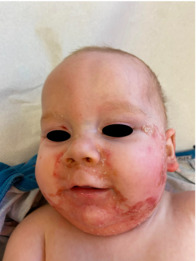

## 1 CASE REPORT

**FIGURE 1:**
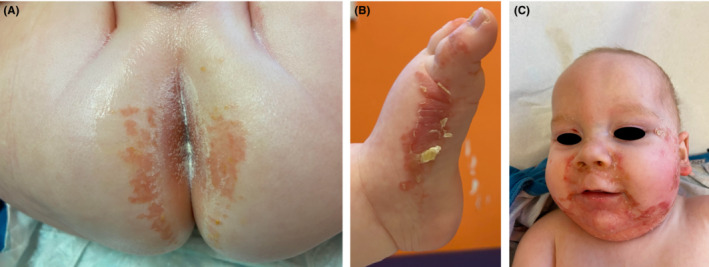
Extensive scaly dermatitis affecting (clockwise from top left) buttocks (A), medial foot (B), and perioral and perinasal skin (C).

**FIGURE 2:**
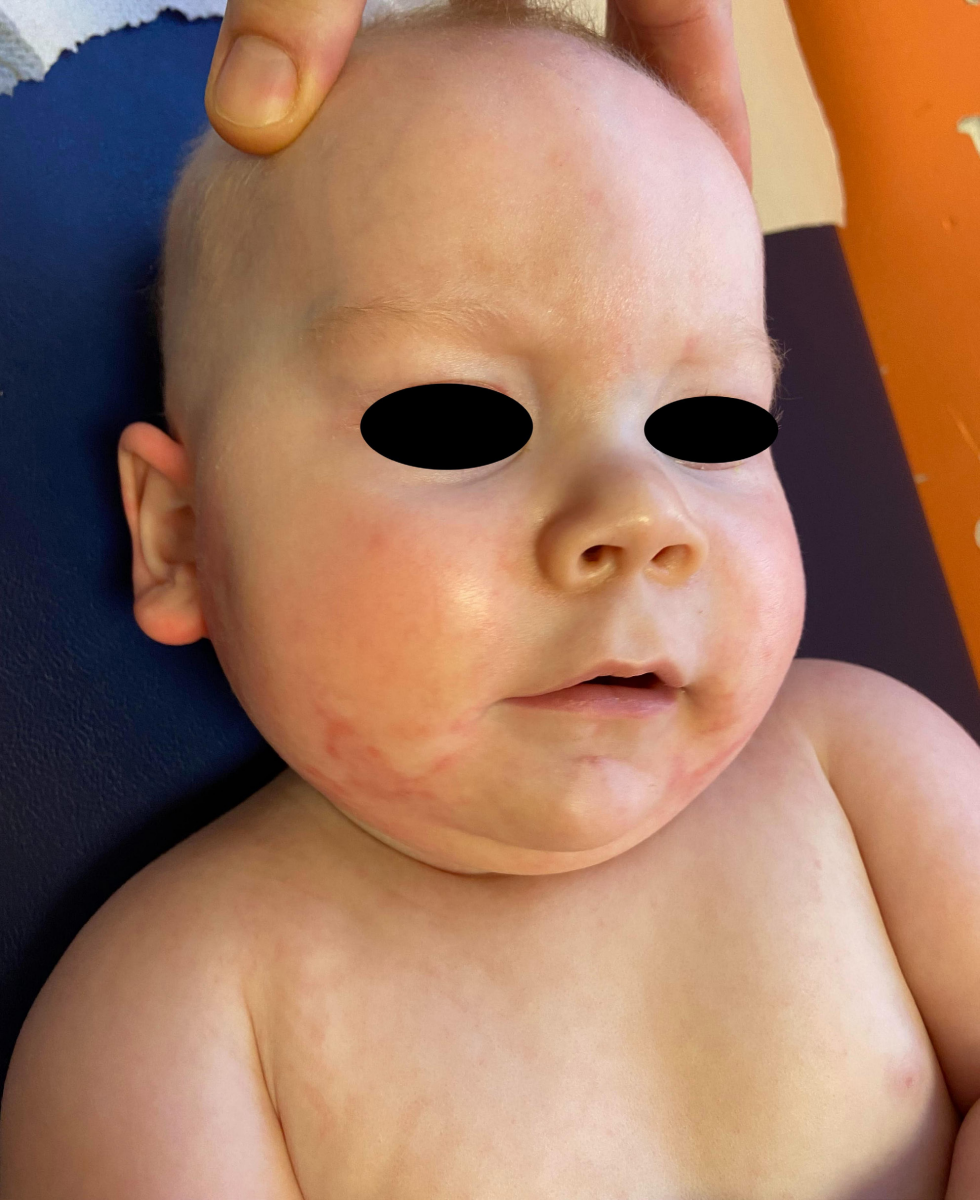
Almost complete resolution of dermatitis within 2 weeks of initiation of zinc supplementation.

A 5‐month‐old male infant presented with a 3‐month history of a progressively worsening scaly eruption associated with recurrent infections, increasing lethargy, poor feeding, and hoarse cry. He had been treated for suspected impetigo with multiple oral antibiotics (amoxicillin, flucloxacillin, and co‐trimoxazole). He was born at term, was exclusively breastfed, and had no family history of skin disease. A dramatic periorifacial, diaper‐area, and acral dermatitis was noted (Figure 1) with loss of occipital hair and thinning of eyelashes. Serum alkaline phosphatase was 41 U/L (normal range 82–383 U/L) and zinc levels were undetectable at <3 μmol/L (normal range 10–25 μmol/L). Maternal breastmilk zinc levels were low (3.15 μmol/L, control mean 12.7 μmol/L) and maternal serum zinc was normal. A rapid improvement was noted within days of starting 3 mg/kg/day zinc sulfate supplementation (Figure 2). Zinc supplementation was stopped after 3 months, with normal follow‐up zinc levels on cessation, following weaning.

Maternal genetic testing for pathogenic variants in *SLC30A2*, a zinc transporter in mammary tissue, detected a variant c.927G>C, resulting in the substitution of tryptophan for cysteine at amino acid position 309. This variant has an allele frequency of <0.01% and in silico tools predict that it is pathogenic. The infant's mother had been prescribed omeprazole 20 mg once daily from 30 weeks' gestation to birth to treat gastro‐esophageal reflux (GER). In her previous pregnancy there was no proton pump inhibitor (PPI) ingestion, and no manifestation of zinc deficiency in the older sibling, who had also been exclusively breastfed.

Infantile zinc deficiency is a rare condition presenting within the first 6 months with periorificial and acral polymorphic and/or erosive crusted plaques. While acrodermatitis enteropathica involves recessive loss‐of‐function pathogenic variants in *SLC39A4*, acquired transient infantile zinc deficiency (TIZD) can be due to prematurity, low breastmilk zinc levels or malnutrition, or malabsorptive processes such as cystic fibrosis.^1^ It is usually rare in breastfed infants due to enhanced bioavailability of zinc.^2^ *SLC30A2* encodes zinc transporter ZnT2, which is responsible for zinc secretion from vesicles in lactating epithelial mammary gland cells.^3^ Homodimer formation between the mutant and wild type causes dysfunction and zinc sequestration in lysosomes of mammary tissue, leading to lower levels in breastmilk.^3^ The mutation in this case has never been previously reported to cause TIZD.^4^ PPI are known to decrease intestinal zinc absorption by increasing intraluminal pH,^5^ as are other medications such as phytates, penicillamine, diuretics, and sodium valproate.^1^ However little is known regarding the effect of these drugs on transplacental or transmammary zinc transmission. Infants may be at increased risk for zinc deficiency and related complications due to increased requirements for zinc in growth and development.^5^

In this case, the affected infant's older sibling had no similar presentation during prolonged exclusive breastfeeding, and the mother had only taken omeprazole for the third trimester of this pregnancy, the critical phase of transplacental zinc transfer in utero.^6^ We hypothesize that antenatal PPI ingestion, in the context of a maternal *SLC30A2* mutation, reduced zinc levels below a threshold that resulted in manifestations of TIZD in this infant. The TIZD in this case also raises concerns about potential nutritional complications of PPI use in infants with physiologic GER.^5^

To our knowledge, this is the first report of this *SLC30A2* mutation associated with TIZD in a breastfed infant, which may have been exacerbated by maternal PPI use during pregnancy, potentially due to diminished transplacental and/or transmammary zinc transmission.

## AUTHOR CONTRIBUTIONS

**Emma Porter:** Investigation; methodology; resources; visualization; writing – original draft; writing – review and editing. **Oonagh Molloy:** Investigation; methodology; supervision; writing – original draft; writing – review and editing. **Michelle Murphy:** Investigation; methodology; supervision; writing – original draft; writing – review and editing. **Cathal O'Connor:** Conceptualization; data curation; formal analysis; investigation; methodology; project administration; resources; software; supervision; validation; visualization; writing – original draft; writing – review and editing.

## CONFLICT OF INTEREST STATEMENT

The authors declare no conflicts of interest.

## CONSENT

Written informed consent was obtained from the patient's parent to publish this report in accordance with the journal's patient consent policy.

## Data Availability

Available on request.
